# Potential drivers of *Megninia ginglymura* (Mégnin) distribution in poultry hens: rearing-system and oviposition microhabitat factors across humid and semi-arid regions

**DOI:** 10.1007/s11259-026-11300-6

**Published:** 2026-06-03

**Authors:** Iasmyn Vitória Cavalcanti Galvão, Maria Beatriz Gomes Duarte, Juan Sebastián D. Cáceres, Lídia Rafaele Almeida da Silva, Antônio Almeida Paz-Neto, Wilton Pires da Cruz, Noeli Juarez Ferla, José Wagner da Silva Melo

**Affiliations:** 1 Universidade Federal de Pernambuco, Recife, PE Brazil; 2 Universidade Federal do Ceará, Fortaleza, CE Brazil; 3 Universidade Federal Rural da Amazônia, Parauapebas, PA Brazil; 4 Universidade do Vale do Taquari, Lajeado, RS Brazil

**Keywords:** Feather mites, Ectoparasites, Spatial distribution, Tropical poultry

## Abstract

*Megninia ginglymura* (Mégnin, 1877) (Acariformes: Analgoidea: Analgidae) is a common feather mite of laying hens and a potential contributor to plumage damage and reduced productivity, yet its population dynamics and microhabitat use remain insufficiently characterized under modern commercial conditions. We conducted a year-long assessment of *M. ginglymura* abundance and intra-host distribution across three major production systems (Automated, Californian (two localities), and Cage-free), situated along a tropical–semi-arid gradient in a major poultry-producing region Pernambuco, Brazil. Monthly sampling of hens revealed pronounced temporal fluctuations in mite abundance, with peak infestations occurring during discrete periods of the production cycle and varying among systems. Cage-free flocks consistently exhibited the lowest infestation levels, whereas Automated and Californian systems showed higher abundances during the early months of monitoring. Female mites were evenly distributed across the five body regions examined, but eggs showed a strong and statistically supported concentration on wing feathers in all systems. This represents the first quantitative demonstration of spatially structured oviposition in *M. ginglymura*, revealing a clear mismatch between female distribution and egg deposition. These results have both ecological and applied implications: they suggest active microhabitat selection during oviposition and identify the wing region as a particularly sensitive diagnostic target for surveillance. By integrating temporal dynamics, housing effects, and spatial organization on the host, this study advances the understanding of *M. ginglymura* ecology and provides practical guidance for monitoring strategies in modern laying hen production.

## Introduction

Feather mites of the genus *Megninia* Berlese, 1883 (Acariformes: Analgoidea: Analgidae) are among the most widespread ectosymbionts associated with domestic poultry, forming a persistent component of the plumage-associated arthropod community across diverse production systems worldwide (Sulzbach et al. 2022; Sparagano et al. 2022; Nahal et al. 2026). According to Gaud et al. (1985), at least four species of the genus *Megninia — M. dipeltata* (Gaud, 1985), *M. cubitalis* (Mégnin, 1877), *M. crinita* (Gaud, 1985), and *M. ortari* (Gaud, 1985) — have been reported in wild individuals of *Gallus gallus*. While many feather mites are traditionally regarded as commensal (Blanco et al. 2001; Galván et al. 2012; Doña et al. 2019), the species *Megninia ginglymura* (Mégnin, 1877) is of particular concern due to recurrent associations with feather degradation, irritation, and reduced productivity in commercial flocks (Tucci et al. 2005). Recent surveys have expanded the known geographic range of this species (Sulzbach et al. 2022), including its first documented occurrence in the state of Pernambuco, Brazil (Duarte et al. 2025), underscoring its widespread distribution and capacity to persist across distinct climatic regions.

Despite its global relevance, the ecology of *M. ginglymura* in modern poultry production remains poorly resolved. A recent global synthesis (Sulzbach et al. 2022) highlights major gaps regarding how environmental, managerial, and host-related factors shape the population dynamics of this species of feather mite, emphasizing that most available data are geographically restricted and often limited to short temporal windows. Existing studies from Brazil (Quintero et al. 2010; Faleiro et al. 2015; Rezende et al. 2015; Horn et al. 2017; Souza et al. 2025) and Cuba (Hernández et al. 2006, 2007) illustrate substantial temporal variability in *M. ginglymura* infestations but reveal no clear or consistent association with season, even though these studies were conducted in markedly different environmental contexts. This absence of climatic synchrony, noted repeatedly in the literature, raises the possibility that local management conditions, flock age, or production stage may outweigh external environmental drivers. However, systematic year-round evaluations across distinct production systems remain scarce.

Another important but insufficiently characterized aspect of *M. ginglymura* biology is its intra-host spatial distribution. Previous investigations report heterogeneous patterns, often with higher concentrations of mites or eggs in dorsal or inner-wing covert feathers (Hernández et al. 2007; Horn et al. 2017; Terril and Shultz 2023), yet these trends vary substantially among studies and production systems. As emphasized by Sulzbach et al. (2022), understanding microhabitat use is crucial for designing efficient sampling strategies and detecting early infestation stages, but quantitative data from contrasting housing systems and climatic zones are still limited.

Taken together, the current knowledge base indicates substantial uncertainty regarding how *M. ginglymura* populations fluctuate across time, how they respond to different production systems, and how females and eggs are distributed across host body regions under real commercial conditions, especially in humid and semi-arid climates such as those of northeastern Brazil. Addressing these gaps is essential not only for improving monitoring and diagnostic protocols but also for informing integrated management approaches in both cage-based and cage-free production technologies.

In this context, the present study provides a year-long comparison of *M. ginglymura* in three major commercial production systems (Automated, Californian, and Cage-free), within two climatically distinct poultry-producing regions of northeastern Brazil. We investigate (i) temporal variation in mite abundance, (ii) differences among production systems and localities, and (iii) the spatial distribution of females and eggs across hen body regions. By integrating temporal, spatial, and system-level dimensions, our study advances the understanding of *M. ginglymura* ecology and offers insights particularly relevant for regions where climatic seasonality is weak and conventional assumptions about environmental drivers may not apply. Based on previous studies, we expected that (i) mite abundance would vary over time, with differences between systems or localities concentrated in specific periods rather than remaining constant throughout the year; and (ii) eggs would show a more structured spatial distribution on the host body than adult females.

## Materials and methods

### Study area and poultry production systems

The study was conducted between January and December 2024 in two commercial laying hen farms located in the state of Pernambuco, Brazil: Paudalho (PD), situated in the Atlantic Forest region, and São Bento do Una (SB), located in the Agreste, a transitional zone between the Atlantic Forest and the semi-arid Caatinga. In each municipality, one farm was visited, and two laying systems were evaluated per farm. In Paudalho, we sampled a Vertical Automated system and a Californian semi-automated system (S1), while in São Bento do Una, we evaluated a Californian semi-automated system (S2) and a Cage-free system. These systems represent the main laying hen production models used throughout Brazil, making the results broadly representative of national commercial practices. Because production systems differed in multiple operational and biological characteristics (e.g., flock size, genetic line, and management), these factors were not disentangled statistically, and comparisons should be interpreted as system-specific rather than causal effects of housing type.

Automated vertical system. Hens were confined in six-tier metal cages. Feed was supplied using automated metal trough feeders, water through nipple drinkers, and eggs were collected by an automated belt. A total of 89,422 Bovans White hens were housed, introduced at 29 weeks of age, and maintained in the system throughout the sampling period.

Semi-automated Californian systems (S1 and S2). Cages were arranged in a stair-step configuration with two stacks per poultry house. Feed and water were provided automatically, whereas egg collection was carried out manually. S1 (Paudalho) housed 6,400 Lohmann LSL hens (≈ 5 hens per cage), introduced at 36 weeks of age, while S2 (São Bento do Una) housed 1,100 Lohmann Brown hens (≈ 4 hens per cage), also introduced at 36 weeks of age.

Cage-free system. Hens were maintained without cages on a sawdust-covered floor, with wooden nest boxes lined with sawdust. The system housed 1,100 Brown Nick hens, introduced at 32 weeks of age. Water was supplied automatically, while feed distribution and egg collection were manual.

### Feather sampling

Sampling was performed monthly. In each poultry house, ten hens were randomly selected monthly. From each bird, two feathers were collected from each of five body regions: breast, rear body (lower tail coverts), back, lower wing covers (wing bar or speculum), and neck (Allonby and Wilson 2019). Feathers were stored in plastic vials containing 70% alcohol. In the laboratory, the vials were shaken to facilitate the removal of mites from the feathers. The alcohol was filtered using 12.5-cm qualitative filter paper (80 g/m²). Mites retained on the filter paper were extracted under a stereomicroscope using a fine-tipped brush. Feathers were subsequently examined directly to quantify egg abundance. Eggs were counted on one half of each feather, and this value was multiplied by two to estimate the total number of eggs per feather.

### Slide preparation and identification

All adult female mites from each of the 5 feather microhabitats (breast, rear body (lower tail coverts), back, inner wing (coverts), and neck were mounted separately on different slides in Hoyer’s medium (Krantz and Walter 2009). Immature stages were not selected due to the difficulty in differentiating them, especially when referring to protonymphs and tritonymphs. Adult females were selected based on their reproductive potential and their contribution to population growth.

Slides were cured at 50–60 °C for 10 days to ensure proper clearing and leg extension. Between 50 and 100 slides were prepared per system per sampling event. Specimens were examined using optical microscopy and identified to species level. Voucher specimens were deposited in the acarological collection at UFPE.

### Abundance of *Megninia ginglymura* in laying systems

To evaluate spatial and temporal patterns in mite abundance, data were partitioned into three analytical subsets, each containing only two levels of the main factor to maintain balanced comparisons: (i) Paudalho: Automated versus Californian (S1); (ii) São Bento do Una: Californian (S2) versus Cage-free; and (iii) Californian systems in different municipalities: S1 (Paudalho) versus S2 (São Bento do Una).

Monthly sampling (12 months × 10 hens per system) allowed testing for effects of System (or Locality), Month, and their interaction. To maintain balanced comparisons, analyses were conducted separately for three subsets: (i) Paudalho: Automated vs. Californian (S1); (ii) São Bento do Una: Californian (S2) vs. Cage-free; and (iii) Californian systems across localities (S1 vs. S2). We initially fitted generalized linear mixed models using glmmTMB package in R, with a negative binomial error distribution (nbinom1) and including Hen as a random intercept to account for potential non-independence: Abundance ∼ System (or Locality) * Month + (1|Hen). Model assumptions were evaluated using the DHARMa package, including tests for overdispersion and zero-inflation. When zero inflation was detected, models were refitted using ziformula = ~ 1. No evidence of overdispersion was detected in the final models. Significance of fixed effects was assessed using Type III Wald χ² tests (car:: Anova). When the interaction term was significant, estimated marginal means were computed using emmeans, followed by adjusted pairwise comparisons within each month. When the interaction was not significant, contrasts were calculated for main effects only.

### Spatial distribution of females and eggs on the host body

**Fig. 1:**
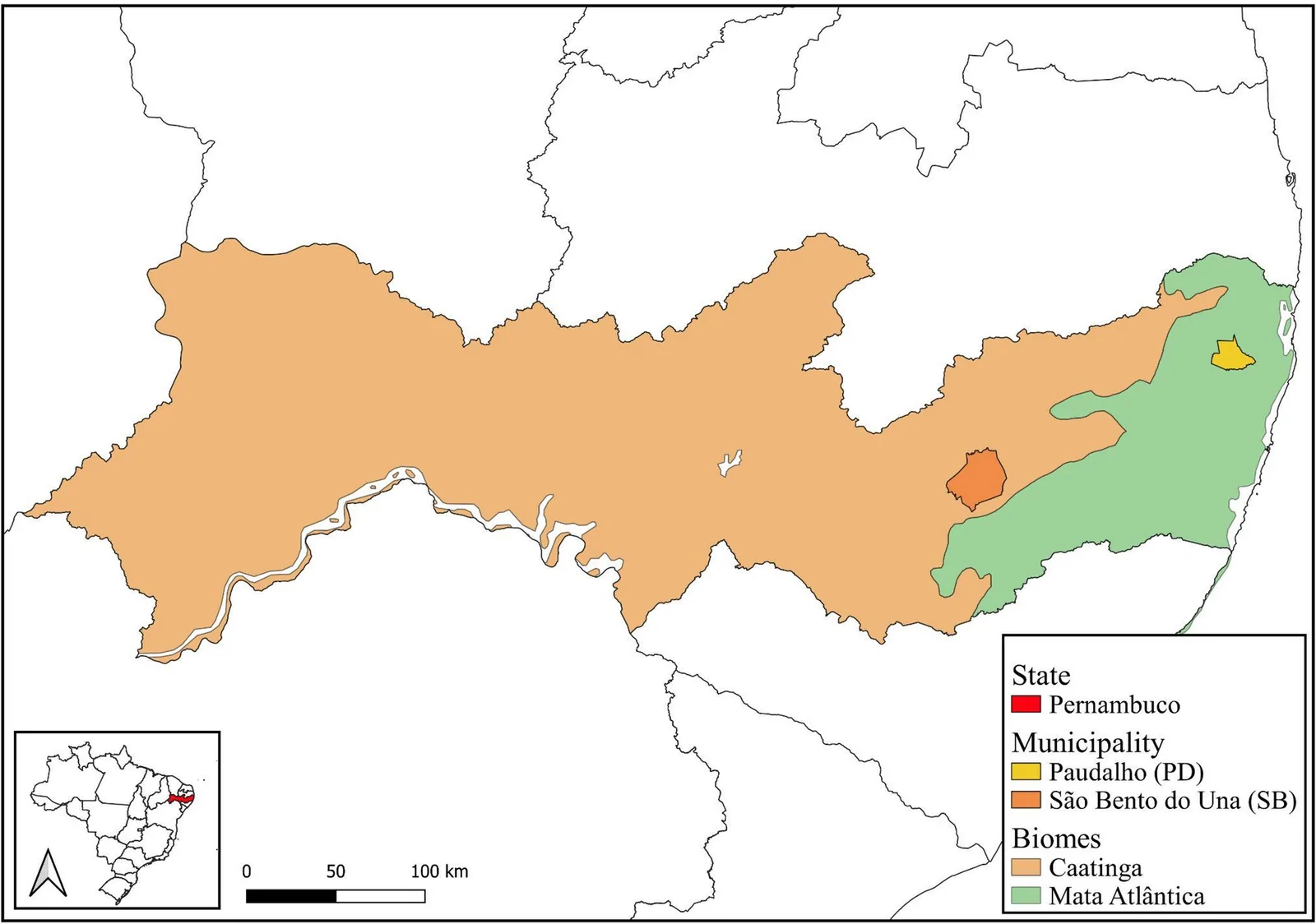
Location of selected commercial laying hen farms and classification of the biomes located in these areas

For each sampled hen, the number of female mites and eggs of *M. ginglymura* was recorded across the five body regions (breast, rear body, back, inner wing, neck) to assess spatial segregation and potential oviposition preference. The spatial distribution of mites was evaluated separately for females and eggs using Pearson’s Chi-square tests of independence, comparing observed frequencies among the five body regions. When significant, standardized residuals were examined to identify which regions contributed disproportionately to deviations from random distribution (Fig. 1).

## Results

### Abundance of *Megninia ginglymura *across laying systems

**Fig. 2:**
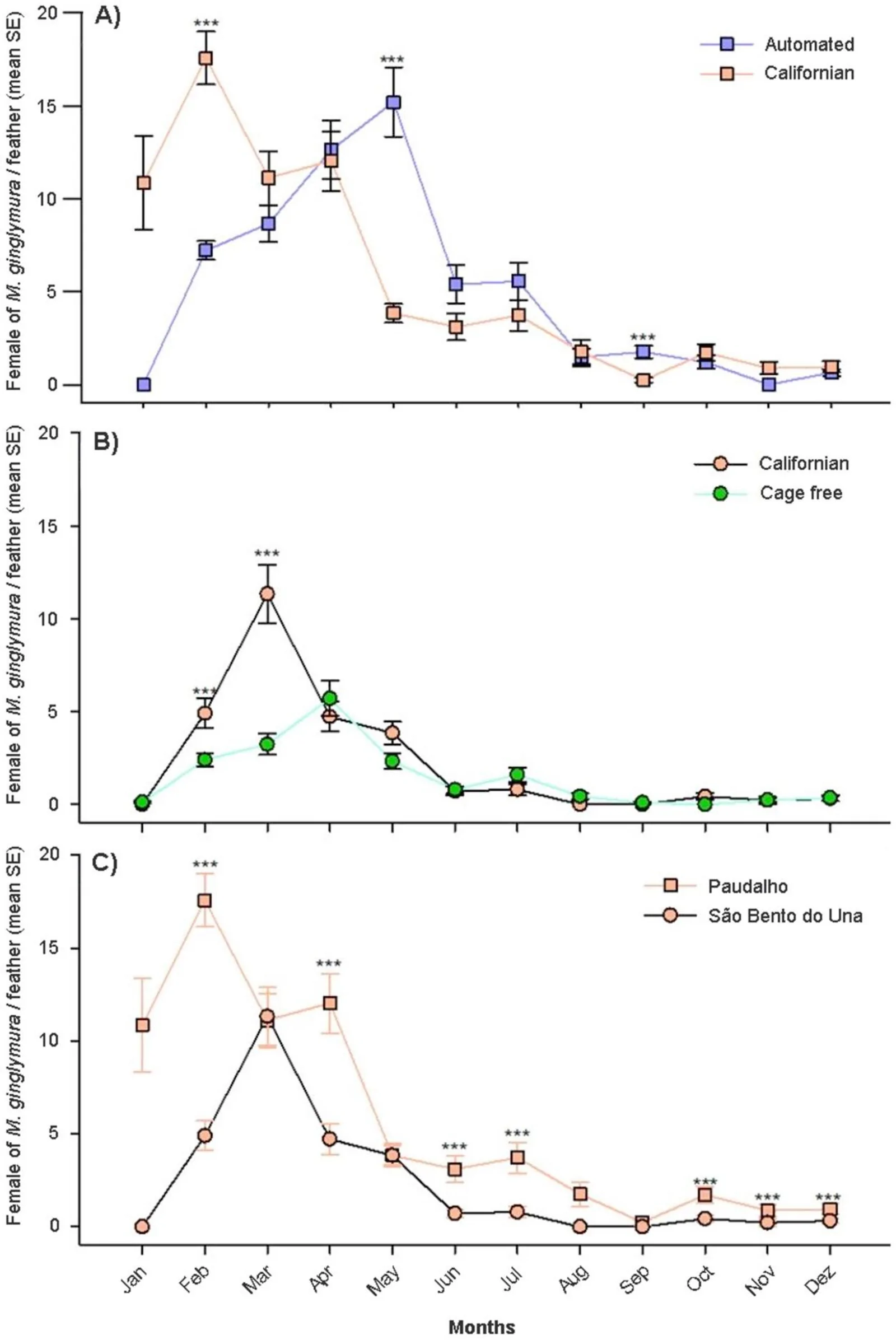
**A-C** Abundance of *Megninia ginglymura* across laying systems. (**A**) Automated and Californian systems from Paudalho (PD). (**B**) Californian and Cage-free systems from Saão Bento do Una (SB). (**C**) Locality comparison between Californian systems. Asterisks *** above the bars denote a highly significant difference (*p* < 0.0001)

The abundance of *M. ginglymura* differed between systems within each locality depending on the month, as indicated by a significant System-Month interaction (χ² = 27.41; df = 11; *P* = 0.003). This interaction is visually evident in Paudalho locality (Fig. 2A), where the Californian system showed markedly higher female abundance in February (z = 3.00; *P* < 0.001), while Automated system hens showed higher values in May (z = 4.59; *P* < 0.001) and September (z = 4.72; *P* < 0.001). No significant differences between systems within this locality were observed from June onward (*P* > 0.29), consistent with the overall seasonal decline detected in both systems. Across months, abundance peaked between February and May (≈ 15–18 mites/feather) and sharply decreased after May, reaching < 2 mites/feather by August and remaining low until December.

In São Bento do Una, where the comparison involved the Californian and Cage-free systems (Fig. 2B), mite abundance was also shaped by a significant System-Month interaction within this locality (χ² = 22.76; df = 11; *P* = 0.019), again indicating that differences between systems varied over time. Female abundance peaked in March to April (≈ 10–12 mites/feather for Californian and ≈ 5–7 for Cage-free), followed by a sustained decline to < 2 mites/feather after July. Differences between the systems within this locality were observed in February (z = 2.18; *P* = 0.0297) and March (z = 3.89; *P* = 0.0001), where the Californian system showed greater abundance, especially in March. In all other months, both systems exhibited similarly low infestation levels within this locality, with no significant differences (*P* > 0.05).

A similar temporal structure characterized the comparison between localities under the same production system (Californian), where female abundance varied markedly through the year but with localities exhibiting distinct seasonal amplitudes (Fig. 2C). The statistical analysis indicated a significant Locality-Month interaction (χ² = 24.47; df = 11; *P* = 0.0109), demonstrating that the difference between localities for the same system (Californian) in Paudalho and São Bento do Una depended on the month. Paudalho consistently exhibited higher abundances during the first half of the year, with significant contrasts in February (z = 3.90; *P* = 0.0001), April (z = 2.85; *P* = 0.0044), June (z = 3.94; *P* = 0.0001), and July (z = 4.24; *P* < 0.0001). Additional but smaller differences occurred in October (z = 3.42; *P* = 0.0006), November (z = 2.99; *P* = 0.0028), and December (z = 2.55; *P* = 0.0107). São Bento do Una showed a higher abundance in March, a result that did not differ from the abundance found in Paudalho in the same month. Despite these differences in magnitude, both localities followed the same overall seasonal decline, with abundances falling to < 1 mite/feather from September onward. The absence of a main effect of Locality (χ² < 0.001; *P* = 0.995) reflects that differences were not constant over time, but instead varied across specific periods of the production cycle.

### Spatial distribution of females and eggs on the host body

**Fig. 3:**
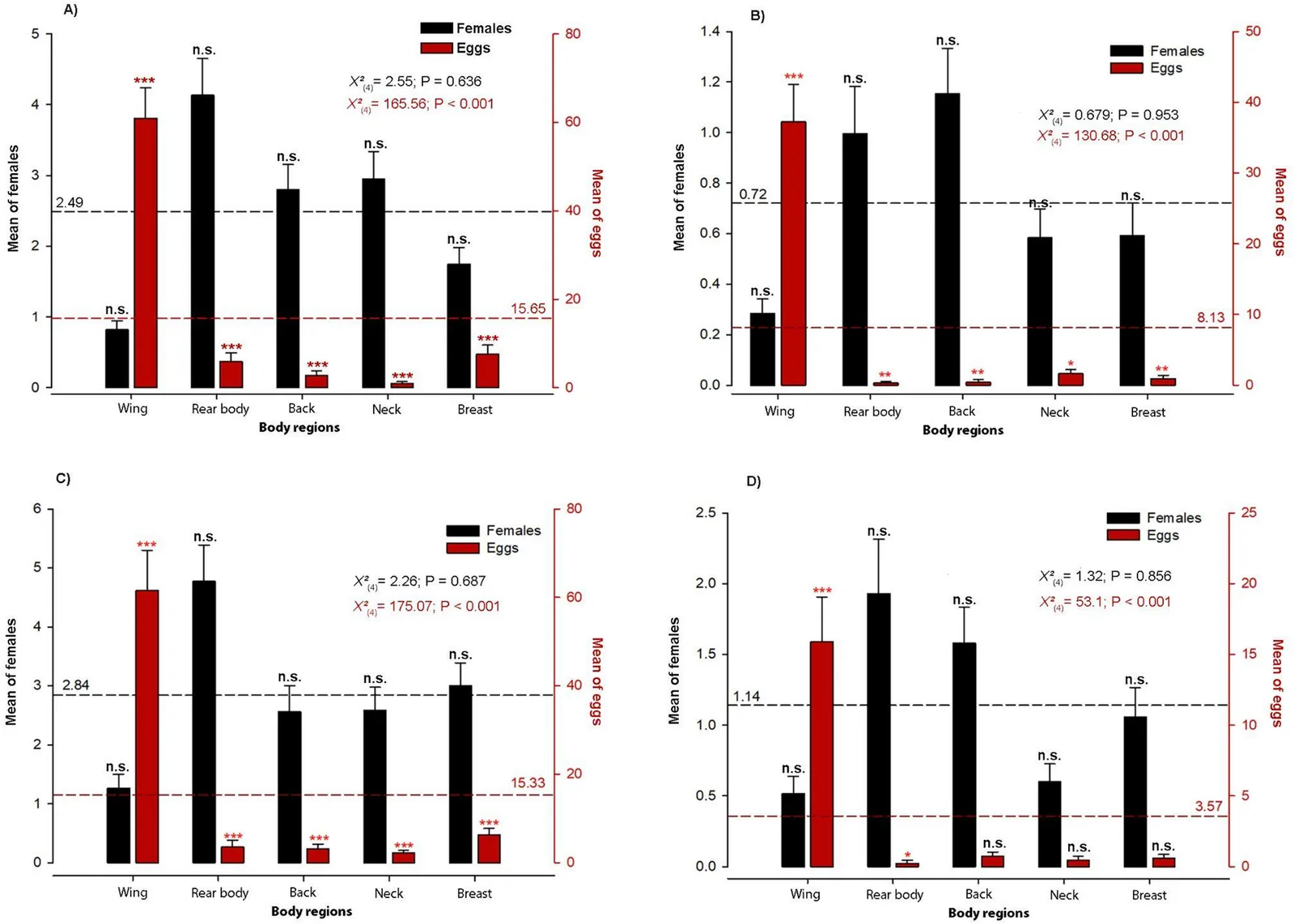
**A-D** Pearson’s Chi-square test for spatial distribution of females and eggs on the host body in different poultry-rearing conditions. **(A)** Automated system, Paudalho (PD). **(B)** Cage-free, São Bento do Una (SB). **(C)** Californian system (PD). **(D)** Californian system (SB). (*p* < 0.0001). Asterisks *** above the bars denote a highly significant difference and **n.s.** not significant

The intra-host distribution of female *M. ginglymura* did not differ among the five sampled body regions in any production system (Californian–Paudalho: χ² = 2.26; df = 4; *P* = 0.6876. Californian–São Bento: χ² = 1.32; df = 4; *P* = 0.8577, Automated: χ² = 2.55; df = 4; *P* = 0.6358, Cage-free: χ² = 0.68; df = 4; *P* = 0.9538) (Fig. 3). Although some body regions, particularly the rear body or back, showed slightly higher mean counts in certain systems, these differences were numerical only and did not reach statistical significance.

In contrast, the distribution of eggs varied among body regions for all production systems (Californian–Paudalho: χ² = 175.07; df = 4; *P* < 0.0001, Californian–São Bento: χ² = 53.10; df = 4; *P* < 0.0001, Automated: χ² = 165.56; df = 4; *P* < 0.0001; Cage-free: χ² = 130.68; df = 4; *P* < 0.0001) (Fig. 3). Across systems, the inner wing consistently exhibited the highest mean egg counts, while the rear body, back, neck, and breast presented much lower values.

## Discussion

*Megninia ginglymura* exhibited marked temporal variation in abundance across the evaluated laying systems, with significant differences between systems within each locality occurring only in specific months. Regarding intra-host distribution, female mites were relatively evenly distributed across the five sampled body regions, whereas eggs showed a heterogeneous pattern, with consistently higher counts on the inner wing region. Together, these findings provide a comprehensive overview of infestation dynamics of *M. ginglymura* across the evaluated production systems and host body regions in commercial laying hens.

The assessment of the population dynamics of *M. ginglymura* in the different areas and systems studied revealed a pattern of greater abundance in the first half of the year (peak between January and May), followed by a gradual population decline in the second half. This result indicates that factors external to the systems, regardless of location, must influence the occurrence of these mites in chickens. The abundance of *M. ginglymura* in the Californian and Automated systems showed a very similar trend, with an average ranging from 10 to 15 mites per feather in the first half of the year, and in the second half an average ranging from 1 to 5 mites per feather. However, in the cage-free system, the abundance of female *M. ginglymura* mites in the first half of the year was more subtle, with a peak that did not exceed 5 mites per feather. Therefore, this last system proved unfavorable to the occurrence of these parasitic mites in chickens. Cage systems impede hens’ performance of natural behaviors important for their welfare such as foraging, perching, nest location inspection, dust bathing, and wing flapping (Rodenburg et al. 2022). Birds require these innate grooming behaviors as a defense against parasitic organisms (Bush & Clayton 2018). In cage free production systems, hens can make choices according to their needs and desires, which is in accordance with welfare definitions (Hartcher & Jones 2017). Dust bathing reduces excess lipids on hens’ feathers and helps maintain their integument in good condition (Olsson & Kelling 2005), and this behavior by laying hens has been shown to help remove ectoparasites (Martin & Mullens 2012). Therefore, the ability of chickens to perform these innate behaviors in cage-free systems could justify the lower abundance of *M. ginglymura* in that system. Although our results reveal clear temporal patterns and system-specific differences in the abundance of *M. ginglymura*, it is important to interpret these findings in light of the study design. Each production system was represented by a single poultry house within each locality, meaning that system and house effects are inherently confounded. Consequently, the observed differences should be interpreted as system-specific patterns within the sampled farms, rather than as generalizable effects across all production systems. This limitation is common in studies conducted under commercial conditions, where replication of large-scale production units is often not feasible.

The temporal fluctuation observed in our study, with infestation peaks occurring in the first half of the year, is similar to previous investigations on *M. ginglymura* in commercial laying hens. For example, Quintero et al. (2010) found a single well-defined peak occurring at the beginning of the year (May). Previous studies indicates no consistent association between *M. ginglymura* abundance and climatic seasonality. Instead, each documents infestation peaks arising during specific temporal windows of the production cycle. For example, Horn et al. (2017) reported two distinct peaks, in March and September, whereas Faleiro et al. (2015) recorded a marked increase in February followed by a second rise in June. These peaks so far apart may be related to the use of mite control methods, but this was not evaluated in these studies. Studies conducted elsewhere reinforce this pattern: Hernández et al. (2006) recorded high prevalence and fluctuating infestation levels in Cuban laying farms, with temporal peaks that did not align with seasonal climatic markers, and Hernández et al. (2007) likewise reported pronounced but irregular increases in mite counts that varied among flocks. Taken together, these studies indicate that *M. ginglymura* populations do not follow a predictable seasonal cycle. Instead, their abundance appears to fluctuate according to factors intrinsic to flock management and production dynamics, although these factors were not directly measured in the present study. Therefore, although previous studies have reported inconsistent associations between climate and mite abundance, the role of climatic variables cannot be assessed in the present study, and no conclusions regarding their predictive value can be drawn from our data. Microhabitat conditions, such as temperature of the host level, may also play an important role, although this hypothesis requires direct evaluation.

Although climatic variables were not directly measured in this study, it is possible to propose biologically plausible hypotheses to explain the observed temporal dynamics based on regional climatic patterns. Mites, like all poikilothermic organisms, rely on external heat sources to regulate their body temperature and sustain essential physiological functions (Gillooly et al. 2002; Woods et al. 2003). Besides, mites are particularly vulnerable to humidity changes (GAEDE & KNÜLLE 1987). Thus, monthly variations in temperature and relative humidity could influence the abundance of *M. ginglymura* throughout the year. In Pernambuco, the first months of the year are characterized by relatively high temperatures and increased rainfall, particularly in the Zona da Mata region (Paudalho), with an average temperature and rainfall of 26 °C and 152 mm between February and April (Pernambuco Water and Climate Agency -APAC 2026) and the AGRITEMPO (2026) website, conditions that are known to favor the development, survival, and reproductive performance of mites. In contrast, the subsequent decline in mite abundance coincides with periods of reduced rainfall, especially in the Agreste region (São Bento do Una), with average of 55 mm between May and September (Pernambuco Water and Climate Agency - APAC 2026) and the AGRITEMPO (2026) website. Mites are highly susceptible to desiccation, and reductions in ambient humidity can negatively affect water balance, survival, and reproductive success. Therefore, the observed decrease in abundance during the second half of the year may reflect suboptimal microclimatic conditions for population maintenance. Under such conditions, egg viability and juvenile development rates may be enhanced, contributing to the population peaks observed in the first half of the year. These interpretations remain hypothetical, as microclimatic conditions at the host level were not directly measured, but they are consistent with known physiological constraints of mites and with the general climatic patterns of the study region.

Differences among production systems within each locality were restricted to specific periods of the monitoring timeline, rather than reflecting persistent or generalizable system-level effects. Notably, hens housed in the Cage-free system consistently exhibited the lowest infestation levels throughout the study, reinforcing the tendency, observed in previous surveys, that more open systems may maintain reduced populations of *M. ginglymura* (Horn et al. 2016; Rezende et al. 2015). In Paudalho and São Bento do Una, both Automated and Californian systems showed higher mite counts during the initial months of sampling, but these contrasts diminished as abundance declined to uniformly low levels across the evaluated systems. Previous studies have shown that factors such as cage density, cage design, or confinement intensity do not reliably predict infestation levels (Rezende et al. 2015), and the limited system-specific patterns observed here align with this conclusion. Furthermore, in our study, only the environment was treated with chemicals (Colosso^®^ Pulverização) during the sanitary break period prior to the arrival of a new batch. Although this practice has been identified as an important driver of mite suppression in earlier studies (Horn et al. 2017), it is not possible to determine whether chemical interventions contributed to the observed decline. Still, the overall low-to-moderate infestation levels recorded across systems are consistent with patterns reported in Brazilian and Cuban farms (Hernández et al. 2006; Rezende et al. 2015), indicating that *M. ginglymura* persists at detectable but generally moderate densities across diverse management contexts, with the Cage-free system standing out as the least affected.

Female mites were distributed uniformly across the five examined body regions, with no statistically significant concentration in any region. Although the cloacal region tended to show numerically higher counts, this trend was not supported statistically. These findings differ from the strong dorsal preference reported by Horn et al. (2017) and Hernández et al. (2007), particularly in farms with high mite abundance. One plausible explanation is that body-region preferences may become more pronounced as total infestation levels rise, whereas at lower densities (such as those observed in our study, maximum of 15–18 mites/feather in the most affected systems) the mites may remain more diffusely distributed across available microhabitats. Such context-dependent spatial patterns have been noted in previous evaluations where systems with greater mite loads exhibited more distinct microhabitat structuring (Horn et al. 2017).

A striking result of this study was the pronounced spatial segregation between female *M. ginglymura* and their eggs. Whereas females were evenly distributed across all examined body regions, eggs were consistently and significantly concentrated on the wing feathers in every production system. This pattern, although previously suspected, had never been quantified or statistically demonstrated. Earlier studies only noted qualitatively that feathers from the inner wing often contained many eggs (Hernández et al. 2007; Horn et al. 2017), but none applied standardized sampling or inferential analyses to confirm oviposition biases. Our results, therefore, provide the first formal, robust quantitative evidence that oviposition in *M. ginglymura* is spatially structured and not merely a by-product of female distribution.

Ecologically, this mismatch between the uniform distribution of females and the highly aggregated distribution of eggs is consistent with the hypothesis of active microhabitat selection during oviposition. Wing feathers may provide structural or microenvironmental features that favor egg retention; however, this interpretation remains hypothetical and requires experimental validation. The behavioral role of hens, particularly grooming, cannot be excluded and should be tested in future experimental studies. The consistency of wing-biased oviposition across housing systems, flock sizes, and distinct climatic regions suggests that this concentration may reflect intrinsic features of mite–host interactions rather than context-specific environmental conditions.

From a practical standpoint, the strong wing bias has major implications for monitoring. Because egg counts responded more clearly than female counts to spatial structuring, even under low infestation intensities, targeting the wing feathers may substantially increase sensitivity for early detection and improve the reliability of surveillance protocols. Given that eggs represent immediate reproductive output, focusing sampling effort on this region may allow earlier identification of population upswings, strengthening integrated management strategies across different production systems.

Together, these results provide an integrated view of temporal variation, system-specific differences within the evaluated farms, and intra-host spatial patterns of *M. ginglymura* in commercial laying hens. Although the clinical impact of this species remains insufficiently understood, previous reports have linked *Megninia* infestations to feather damage, irritation, and reduced egg production (Tucci et al. 2005). The present findings confirm that the species is widespread across the evaluated commercial systems in Pernambuco and that peak periods may generate local increases in abundance even in the absence of sustained high infestation levels. Because egg deposition was consistently concentrated on the wing, this region may offer a more sensitive diagnostic target during routine monitoring, particularly in systems with low overall mite abundance.

## Data Availability

All data can be found at the following link: https://doi.org/10.5281/zenodo.18756627.
